# Chlordiazepoxide dichloro­methane monosolvate

**DOI:** 10.1107/S1600536812009695

**Published:** 2012-03-10

**Authors:** Andreas Fischer

**Affiliations:** a Division of Applied Physical Chemistry, School of Chemical Science and Engineering, 100 44 Stockholm, Sweden

## Abstract

In the title compound (systematic name: 7-chloro-2-methyl­amino-5-phenyl-3*H*-1,4-benzodiazepine 4-oxide dichloro­meth­ane monosolvate), C_16_H_14_ClN_3_O·CH_2_Cl_2_, the seven-membered ring adopts a boat conformation with the CH_2_ group as the prow and the two aromatic C atoms as the stern. The dihedral angle between the benzene rings is 75.25 (6)°. The crystal structure features centrosymmetric pairs of chlordiazepoxide mol­ecules linked by pairs of N—H⋯O hydrogen bonds, which generate *R*_2_^2^(12) loops.

## Related literature

For the synthesis of chlordiazepoxide, see: Sternbach *et al.* (1961 ▶). For the structure of chlordiazepoxide, see: Bertolasi *et al.* (1982 ▶). For the structure of a second polymorph of chlordiazepoxide, see: Singh *et al.* (1998 ▶). For the structure of chlordiazepoxide hydro­chloride, see: Herrnstadt *et al.* (1979 ▶). For the early history of benzopdiazepines, see: Sternbach (1979 ▶).
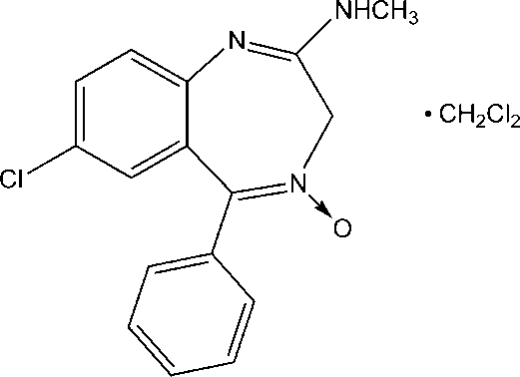


## Experimental

### 

#### Crystal data

C_16_H_14_ClN_3_O·CH_2_Cl_2_
*M* *_r_* = 384.69
Triclinic, 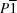

*a* = 7.8310 (12) Å
*b* = 9.461 (2) Å
*c* = 12.6947 (5) Å
α = 94.284 (11)°
β = 93.821 (9)°
γ = 108.499 (13)°
*V* = 885.4 (2) Å^3^
*Z* = 2
Mo *K*α radiation
μ = 0.53 mm^−1^
*T* = 173 K
0.60 × 0.33 × 0.04 mm

#### Data collection

Bruker–Nonius KappaCCD diffractometer
Absorption correction: multi-scan *SADABS* (Sheldrick, 2003 ▶) *T*_min_ = 0.763, *T*_max_ = 0.979
20348 measured reflections
4040 independent reflections
3124 reflections with *I* > 2σ(*I*)
*R*_int_ = 0.034

#### Refinement

*R*[*F* ^2^ > 2σ(*F* ^2^)] = 0.040
*wR*(*F* ^2^) = 0.104
*S* = 1.03
4040 reflections
221 parameters
1 restraint
H atoms treated by a mixture of independent and constrained refinement
Δρ_max_ = 0.76 e Å^−3^
Δρ_min_ = −0.52 e Å^−3^

### 

Data collection: *COLLECT* (Nonius, 1999 ▶); cell refinement: *DIRAX* (Duisenberg, 1992 ▶); data reduction: *EVALCCD* (Duisenberg *et al.*, 2003 ▶); program(s) used to solve structure: *SHELXS97* (Sheldrick, 2008 ▶); program(s) used to refine structure: *SHELXL97* (Sheldrick, 2008 ▶); molecular graphics: *DIAMOND* (Brandenburg, 2007 ▶).; software used to prepare material for publication: *publCIF* (Westrip, 2010 ▶).

## Supplementary Material

Crystal structure: contains datablock(s) global, I. DOI: 10.1107/S1600536812009695/hb6668sup1.cif

Structure factors: contains datablock(s) I. DOI: 10.1107/S1600536812009695/hb6668Isup2.hkl

Supplementary material file. DOI: 10.1107/S1600536812009695/hb6668Isup3.cml

Additional supplementary materials:  crystallographic information; 3D view; checkCIF report

## Figures and Tables

**Table 1:** Hydrogen-bond geometry (Å, °)

*D*—H⋯*A*	*D*—H	H⋯*A*	*D*⋯*A*	*D*—H⋯*A*
N3—H3*A*⋯O1^i^	0.85 (2)	2.08 (2)	2.916 (2)	166 (2)

Symmetry code: (i) 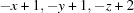.

**Fig. 1.**
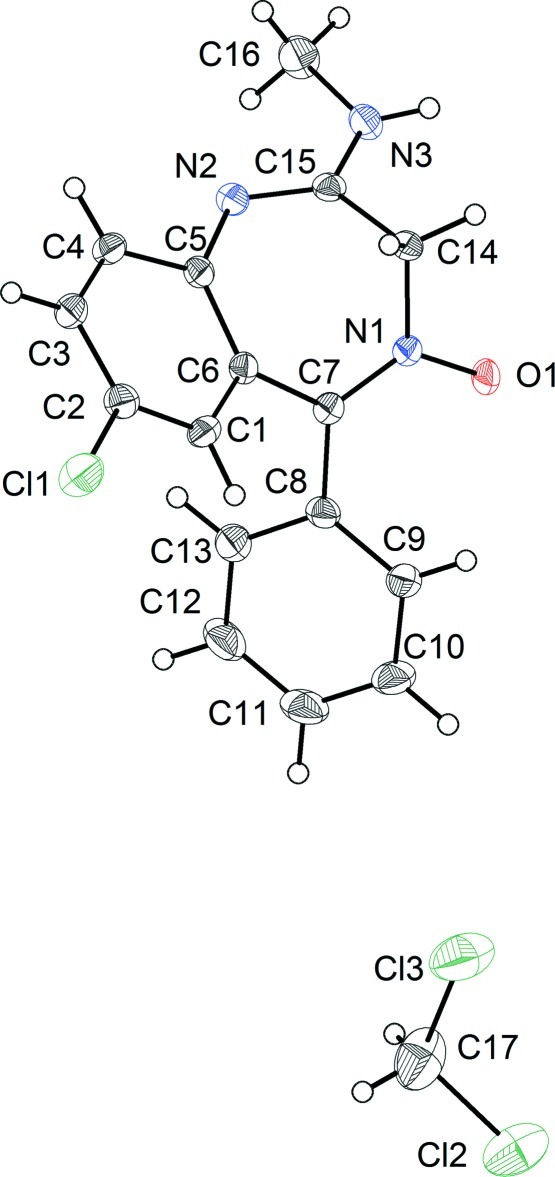
The molecular structure of the title compound. Displacement ellipsoids are drawn at the 50% probability level.

**Fig. 2.**
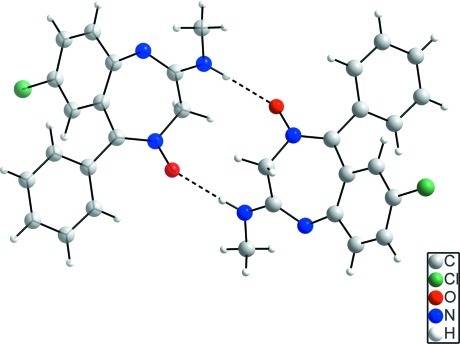
The centrosymmetric chlordiazepoxide dimers in the title compound. The half-transparent moiety is generated by (i)–x+1, –y+1, –z+2.

## supplementary crystallographic information

### Comment

Chlordiazepozide (7-chloro-2-methylamino-5-phenyl-3*H*-1,4-benzodiazepine 4-oxide) was the first benzodiazepine to enter the market as member of a new class of powerful tranquilizers. The crystal structure of the hydrochloride was determined in 1979 (Herrnstadt *et al.*, 1979), the structure of the pure compound in 1982 (Bertolasi *et al.*, 1982). The reason for the study of chlordiazepoxide salts was the presence of three potential protonation sites (Herrnstadt *et al.* 1979). In order to study a number of different chlordiazepoxide salts, we synthesized the compound according to the description by Sternbach *et al.* (1961). The final step in this synthesis is the crystallization from dichloromethane. It turned out that the crystallization product that forms initially is a chlordiazepoxide dichloromethane solvate, whose structure is described here. The title compound (Fig. 1) features pairs of chlordiazepoxide molecules, which are hydrogen-bonded *via* two symmetry-equivalent N–H···O bonds across an inversion centre (Fig. 2). The same pattern could be found in the hydrochloride (Herrnstadt *et al.*, 1979). In the structure of pure chlordiazepoxide, pairs of molecules of the same chirality (due to the high energy barrier of ring inversion, benzodiazepines are chiral) were observed and it was argued that this arrangement might be more stable than dimers of two different enantiomers. However, this effect must be quite subtle since the only interactions between the chlordiazepoxide and dichloromethane molecules are due to van der Waals forces. The dihedral angle between the two phenyl groups is 75.25 (6)°.

### Experimental

Chlordiazepoxide was synthesized according to the procedure described by Sternbach *et al.*(1961), starting from commercial 2-amino-5-chlorobenzophenone. Slow evaporation of the solution of the final product in dichloromethane yielded colourless, block-shaped crystals of the title compound. Once removed from the mother liquor, the crystals decomposed slowly (within days), yielding solvent-free chlordiazepoxide.

### Refinement

All H atoms except that attached to N were placed at calculated positions and refined riding. The N–H atom was located from the Fourier map and refined with the N–H distance restrained to 0.88 (2) Å.

### Crystal data

**Table:**

C_16_H_14_ClN_3_O·CH_2_Cl_2_	*Z* = 2
*M**_r_* = 384.69	*F*(000) = 396
Triclinic, *P*1	*D*_x_ = 1.443 Mg m^−^^3^
Hall symbol: -P 1	Mo *K*α radiation, λ = 0.71073 Å
*a* = 7.8310 (12) Å	Cell parameters from 38 reflections
*b* = 9.461 (2) Å	θ = 8.7–19.5°
*c* = 12.6947 (5) Å	µ = 0.53 mm^−^^1^
α = 94.284 (11)°	*T* = 173 K
β = 93.821 (9)°	Plate, colourless
γ = 108.499 (13)°	0.60 × 0.33 × 0.04 mm
*V* = 885.4 (2) Å^3^	

### Data collection

**Table:**

Bruker–Nonius KappaCCD diffractometer	4040 independent reflections
Radiation source: fine-focus sealed tube	3124 reflections with *I* > 2σ(*I*)
Graphite monochromator	*R*_int_ = 0.034
φ&ω scans	θ_max_ = 27.5°, θ_min_ = 4.5°
Absorption correction: multi-scan *SADABS* (Sheldrick, 2003)	*h* = −10→10
*T*_min_ = 0.763, *T*_max_ = 0.979	*k* = −12→12
20348 measured reflections	*l* = −15→16

### Refinement

**Table:**

Refinement on *F*^2^	Primary atom site location: structure-invariant direct methods
Least-squares matrix: full	Secondary atom site location: difference Fourier map
*R*[*F*^2^ > 2σ(*F*^2^)] = 0.040	H atoms treated by a mixture of independent and constrained refinement
*wR*(*F*^2^) = 0.104	*w* = 1/[σ^2^(*F*_o_^2^) + (0.0409*P*)^2^ + 0.7625*P*] where *P* = (*F*_o_^2^ + 2*F*_c_^2^)/3
*S* = 1.03	(Δ/σ)_max_ < 0.001
4040 reflections	Δρ_max_ = 0.76 e Å^−^^3^
221 parameters	Δρ_min_ = −0.52 e Å^−^^3^
1 restraint	

### Special details

**Table:**

Geometry. All e.s.d.'s (except the e.s.d. in the dihedral angle between two l.s. planes) are estimated using the full covariance matrix. The cell e.s.d.'s are taken into account individually in the estimation of e.s.d.'s in distances, angles and torsion angles; correlations between e.s.d.'s in cell parameters are only used when they are defined by crystal symmetry. An approximate (isotropic) treatment of cell e.s.d.'s is used for estimating e.s.d.'s involving l.s. planes.
Refinement. Refinement of *F*^2^ against ALL reflections. The weighted *R*-factor *wR* and goodness of fit *S* are based on *F*^2^, conventional *R*-factors *R* are based on *F*, with *F* set to zero for negative *F*^2^. The threshold expression of *F*^2^ > σ(*F*^2^) is used only for calculating *R*-factors(gt) *etc*. and is not relevant to the choice of reflections for refinement. *R*-factors based on *F*^2^ are statistically about twice as large as those based on *F*, and *R*- factors based on ALL data will be even larger.

### Fractional atomic coordinates and isotropic or equivalent isotropic displacement parameters (Å^2^)

**Table:**

	*x*	*y*	*z*	*U*_iso_*/*U*_eq_	
C1	0.4054 (3)	0.0142 (2)	0.68015 (14)	0.0228 (4)	
C2	0.4147 (3)	−0.1283 (2)	0.67919 (15)	0.0233 (4)	
C3	0.3620 (3)	−0.2137 (2)	0.76314 (15)	0.0229 (4)	
C4	0.3110 (2)	−0.1489 (2)	0.85106 (14)	0.0219 (4)	
C5	0.3146 (2)	0.00119 (19)	0.85985 (14)	0.0183 (3)	
C6	0.3543 (2)	0.08072 (19)	0.76981 (14)	0.0188 (4)	
C7	0.3392 (2)	0.23116 (19)	0.76213 (14)	0.0193 (4)	
C8	0.2418 (3)	0.2605 (2)	0.66676 (14)	0.0213 (4)	
C9	0.3090 (3)	0.3890 (2)	0.61483 (15)	0.0251 (4)	
C10	0.2113 (3)	0.4097 (2)	0.52552 (16)	0.0335 (5)	
C11	0.0461 (3)	0.3048 (3)	0.48835 (17)	0.0375 (5)	
C12	−0.0217 (3)	0.1780 (3)	0.53961 (18)	0.0374 (5)	
C13	0.0762 (3)	0.1547 (2)	0.62793 (16)	0.0300 (4)	
C14	0.4974 (2)	0.2983 (2)	0.93612 (13)	0.0189 (4)	
C15	0.3509 (2)	0.1940 (2)	0.99178 (13)	0.0186 (4)	
C16	0.1763 (3)	0.1635 (2)	1.14472 (17)	0.0334 (5)	
C17	0.0152 (4)	0.5580 (3)	0.1687 (3)	0.0561 (7)	
Cl1	0.50033 (8)	−0.20239 (6)	0.57295 (4)	0.03756 (16)	
Cl2	0.04988 (9)	0.72192 (8)	0.10404 (6)	0.0542 (2)	
Cl3	0.19167 (10)	0.57040 (9)	0.26233 (6)	0.0620 (2)	
O1	0.39853 (18)	0.47104 (13)	0.84472 (10)	0.0218 (3)	
N1	0.4092 (2)	0.33547 (16)	0.84073 (11)	0.0176 (3)	
N2	0.2700 (2)	0.05504 (17)	0.95575 (12)	0.0203 (3)	
N3	0.3075 (2)	0.25397 (18)	1.08078 (12)	0.0227 (3)	
H1	0.4336	0.0679	0.6199	0.027*	
H3	0.3613	−0.3145	0.7599	0.027*	
H4	0.2720	−0.2076	0.9078	0.026*	
H9	0.4214	0.4621	0.6407	0.030*	
H10	0.2581	0.4963	0.4896	0.040*	
H11	−0.0206	0.3202	0.4275	0.045*	
H12	−0.1357	0.1066	0.5144	0.045*	
H13	0.0303	0.0662	0.6621	0.036*	
H14A	0.5617	0.3906	0.9836	0.023*	
H14B	0.5862	0.2490	0.9159	0.023*	
H16A	0.0613	0.1156	1.1011	0.050*	
H16B	0.1578	0.2274	1.2043	0.050*	
H16C	0.2211	0.0863	1.1720	0.050*	
H17A	−0.0980	0.5380	0.2038	0.067*	
H17B	0.0001	0.4723	0.1151	0.067*	
H3A	0.379 (3)	0.3395 (19)	1.1074 (17)	0.027*	

### Atomic displacement parameters (Å^2^)

**Table:**

	*U*^11^	*U*^22^	*U*^33^	*U*^12^	*U*^13^	*U*^23^
C1	0.0312 (10)	0.0182 (9)	0.0194 (9)	0.0079 (8)	0.0027 (7)	0.0051 (7)
C2	0.0296 (10)	0.0209 (9)	0.0205 (9)	0.0104 (8)	0.0026 (7)	0.0002 (7)
C3	0.0275 (10)	0.0175 (9)	0.0247 (9)	0.0094 (8)	−0.0012 (8)	0.0030 (7)
C4	0.0233 (9)	0.0206 (9)	0.0214 (9)	0.0059 (7)	0.0006 (7)	0.0067 (7)
C5	0.0156 (8)	0.0184 (8)	0.0192 (8)	0.0037 (7)	−0.0011 (7)	0.0015 (6)
C6	0.0206 (9)	0.0148 (8)	0.0200 (9)	0.0049 (7)	−0.0020 (7)	0.0013 (6)
C7	0.0209 (9)	0.0169 (8)	0.0197 (9)	0.0055 (7)	0.0009 (7)	0.0028 (6)
C8	0.0288 (10)	0.0192 (9)	0.0178 (9)	0.0117 (8)	−0.0014 (7)	−0.0008 (7)
C9	0.0343 (11)	0.0206 (9)	0.0209 (9)	0.0100 (8)	0.0001 (8)	0.0017 (7)
C10	0.0522 (14)	0.0285 (10)	0.0247 (10)	0.0199 (10)	−0.0009 (9)	0.0070 (8)
C11	0.0535 (15)	0.0380 (12)	0.0262 (11)	0.0260 (11)	−0.0128 (10)	0.0004 (9)
C12	0.0385 (13)	0.0344 (12)	0.0349 (12)	0.0114 (10)	−0.0164 (10)	−0.0044 (9)
C13	0.0361 (12)	0.0227 (10)	0.0292 (10)	0.0087 (9)	−0.0062 (9)	0.0024 (8)
C14	0.0175 (9)	0.0201 (9)	0.0175 (8)	0.0044 (7)	−0.0028 (7)	0.0023 (7)
C15	0.0166 (8)	0.0221 (9)	0.0165 (8)	0.0058 (7)	−0.0015 (7)	0.0038 (7)
C16	0.0309 (11)	0.0362 (11)	0.0273 (11)	0.0018 (9)	0.0105 (9)	−0.0007 (8)
C17	0.0338 (14)	0.0492 (15)	0.085 (2)	0.0101 (12)	0.0003 (13)	0.0207 (14)
Cl1	0.0618 (4)	0.0276 (3)	0.0305 (3)	0.0216 (2)	0.0185 (2)	0.00376 (19)
Cl2	0.0412 (3)	0.0717 (5)	0.0646 (4)	0.0315 (3)	0.0154 (3)	0.0322 (3)
Cl3	0.0553 (4)	0.0777 (5)	0.0559 (4)	0.0205 (4)	0.0033 (3)	0.0317 (4)
O1	0.0268 (7)	0.0131 (6)	0.0243 (7)	0.0060 (5)	−0.0019 (5)	−0.0003 (5)
N1	0.0189 (7)	0.0153 (7)	0.0183 (7)	0.0051 (6)	0.0001 (6)	0.0026 (5)
N2	0.0203 (8)	0.0206 (8)	0.0185 (7)	0.0048 (6)	0.0013 (6)	0.0018 (6)
N3	0.0214 (8)	0.0231 (8)	0.0203 (8)	0.0036 (7)	0.0013 (6)	−0.0015 (6)

### Geometric parameters (Å, º)

**Table:**

C1—C2	1.372 (3)	C16—N3	1.451 (3)
C1—C6	1.402 (2)	C17—Cl3	1.730 (3)
C2—C3	1.391 (3)	C17—Cl2	1.762 (3)
C2—Cl1	1.7428 (19)	O1—N1	1.3090 (18)
C3—C4	1.376 (3)	C1—H1	0.9500
C4—C5	1.407 (2)	C3—H3	0.9500
C5—N2	1.393 (2)	C4—H4	0.9500
C5—C6	1.413 (2)	C9—H9	0.9500
C6—C7	1.475 (2)	C10—H10	0.9500
C7—N1	1.306 (2)	C11—H11	0.9500
C7—C8	1.478 (2)	C12—H12	0.9500
C8—C13	1.394 (3)	C13—H13	0.9500
C8—C9	1.396 (3)	C14—H14A	0.9900
C9—C10	1.386 (3)	C14—H14B	0.9900
C10—C11	1.384 (3)	C16—H16A	0.9800
C11—C12	1.380 (3)	C16—H16B	0.9800
C12—C13	1.387 (3)	C16—H16C	0.9800
C14—N1	1.474 (2)	C17—H17A	0.9900
C14—C15	1.511 (2)	C17—H17B	0.9900
C15—N2	1.297 (2)	N3—H3A	0.854 (16)
C15—N3	1.338 (2)		
			
C2—C1—C6	120.20 (16)	C6—C1—H1	119.9
C1—C2—C3	121.22 (17)	C4—C3—H3	120.7
C1—C2—Cl1	119.68 (14)	C2—C3—H3	120.7
C3—C2—Cl1	119.07 (14)	C3—C4—H4	118.8
C4—C3—C2	118.55 (16)	C5—C4—H4	118.8
C3—C4—C5	122.33 (16)	C10—C9—H9	120.1
N2—C5—C4	116.32 (15)	C8—C9—H9	120.1
N2—C5—C6	126.13 (16)	C11—C10—H10	119.8
C4—C5—C6	117.48 (16)	C9—C10—H10	119.8
C1—C6—C5	119.75 (16)	C12—C11—H11	120.0
C1—C6—C7	116.61 (15)	C10—C11—H11	120.0
C5—C6—C7	123.60 (16)	C11—C12—H12	120.0
N1—C7—C6	119.34 (16)	C13—C12—H12	120.0
N1—C7—C8	121.14 (15)	C12—C13—H13	119.8
C6—C7—C8	119.51 (15)	C8—C13—H13	119.8
C13—C8—C9	119.24 (17)	N1—C14—H14A	110.2
C13—C8—C7	118.08 (16)	C15—C14—H14A	110.2
C9—C8—C7	122.68 (17)	N1—C14—H14B	110.2
C10—C9—C8	119.88 (19)	C15—C14—H14B	110.2
C11—C10—C9	120.4 (2)	H14A—C14—H14B	108.5
C12—C11—C10	120.04 (19)	N3—C16—H16A	109.5
C11—C12—C13	120.1 (2)	N3—C16—H16B	109.5
C12—C13—C8	120.31 (19)	H16A—C16—H16B	109.5
N1—C14—C15	107.42 (14)	N3—C16—H16C	109.5
N2—C15—N3	121.51 (16)	H16A—C16—H16C	109.5
N2—C15—C14	122.57 (16)	H16B—C16—H16C	109.5
N3—C15—C14	115.91 (16)	Cl3—C17—H17A	109.0
Cl3—C17—Cl2	112.88 (15)	Cl2—C17—H17A	109.0
C7—N1—O1	124.88 (15)	Cl3—C17—H17B	109.0
C7—N1—C14	118.94 (14)	Cl2—C17—H17B	109.0
O1—N1—C14	116.05 (13)	H17A—C17—H17B	107.8
C15—N2—C5	118.83 (15)	C15—N3—H3A	117.0 (15)
C15—N3—C16	121.24 (16)	C16—N3—H3A	119.7 (15)
C2—C1—H1	119.9		

### Hydrogen-bond geometry (Å, º)

**Table:**

*D*—H···*A*	*D*—H	H···*A*	*D*···*A*	*D*—H···*A*
N3—H3*A*···O1^i^	0.85 (2)	2.08 (2)	2.916 (2)	166 (2)

Symmetry code: (i) −*x*+1, −*y*+1, −*z*+2.
